# Effects of Size-Fractionated Particulate Matter on Cellular Oxidant Radical Generation in Human Bronchial Epithelial BEAS-2B Cells

**DOI:** 10.3390/ijerph13050483

**Published:** 2016-05-10

**Authors:** Longfei Guan, Wei Rui, Ru Bai, Wei Zhang, Fang Zhang, Wenjun Ding

**Affiliations:** Laboratory of Environment and Health, College of Life Sciences, University of Chinese Academy of Sciences, Beijing 100049, China; guanlongfei12@mails.ucas.ac.cn (L.G.); ruiwei08@mails.ucas.ac.cn (W.R.); bairu10@mails.ucas.ac.cn (R.B.); zhangw@ucas.ac.cn (W.Z.)

**Keywords:** particulate matter, size-fraction, chemical composition, correlation analysis, cytotoxicity

## Abstract

The aim of the present study was to investigate the effects of size-fractionated (*i.e.*, <1; 1–2.5, and 2.5–10 µm in an aerodynamic diameter) ambient particulate matter (PM) on reactive oxygen species (ROS) activity and cell viability in human bronchial epithelial cells (BEAS-2B). The PM samples were collected from an urban site (uPM) in Beijing and a steel factory site (sPM) in Anshan, China, from March 2013 to December 2014. Metal elements, organic and elemental carbon, and water-soluble inorganic ions in the uPM and sPM were analyzed. The cell viability and ROS generation in PM-exposed BEAS-2B cells were measured by MTS and DCFH-DA. The results showed that both uPM and sPM caused a decrease in the cell viability and an increase in ROS generation. The level of ROS measured in sPM_1.0_ was approximately triple that in uPM_1.0_. The results of correlation analysis showed that the ROS activity and cytotoxicity were related to different PM composition. Moreover, deferoxamine (DFO) significantly prevented the increase of ROS generation and the decrease of cell viability. Taken together, our results suggest that the metals absorbed on PM induced oxidant radical generation in BEAS-2B cells that could lead to impairment of pulmonary function.

## 1. Introduction

In recent years, industrial emissions, urban construction, and increased vehicle exhausts led to poor air quality in many cities in China [1,2]. The adverse health effects of airborne particulate matters (PM) pollution have become a growing concern [1,3]. Numerous epidemiological studies have documented a positive correlation between exposure to ambient PM concentrations and increased respiratory and cardiovascular morbidity and mortality [4,5,6]. However, the contribution of specific components of PM to its toxicity is largely unknown, despite the recognition that the chemical composition of PM is clearly important in driving its toxicological effects.

Generally, the size, shape, and chemical composition of PM play crucial roles in the adverse effects on human health. PM can be classified into different size fractions, such as coarse particles (PM_2.5–10_, aerodynamic diameter less than 10 µm but larger than 2.5 µm), fine particles (PM_2.5_ and PM_1_, aerodynamic diameter <2.5 µm and <1 µm), and ultrafine particles (PM_0.1_, aerodynamic diameter ≤100 nm) [7,8,9]. The penetration depth and deposition of PM in the lung are particle size dependent [10]. Particular attention has been paid to respirable fine particles. It has been reported that PM_2.5–10_ tend to deposit in the nasopharyngeal compartment, while PM_2.5–1_ and PM_0.1_ exhibit deposition in the alveolar and tracheobronchial compartments [11]. These small particles retained in the peripheral lung may be more harmful than larger particles [12,13].

On the other hand, PM-induced toxicity is also affected by the chemical composition and source [14]. PM is a complex mixture of particles with different chemical composition. The coarse fraction is mostly primary particles consisting of crustal elements, sea salt, and organic components [8]. The fine fraction contains a mixture of carbonaceous material derived from anthropogenic emissions, such as inorganic elements, sulfates, nitrates, chloride, ammonium, element carbon, and organic carbonaceous matter [15]. In the city of Beijing (China), traffic emissions are the major source of air pollution. Sulfate, nitrate, ammonium, metals, and organic and element carbon have been found to comprise the major chemical composition of PM_2.5_ in Beijing [16,17]. However, the contribution of individual chemical composition of urban PM to toxicity remains to be elucidated.

Oxidative stress has been recognized as a key molecular mechanism of PM-mediated cytotoxicity [18,19]. Basically, PM-induced oxidative stress is a state of redox disequilibrium in which reactive oxygen species (ROS) production overwhelms the antioxidant defenses, thereby leading to adverse biological consequences [20]. ROS are composed of hydrogen peroxide (H_2_O_2_), superoxide (O_2_^−^), hydroxyl radicals (OH∙), or other hydroperoxides (ROOH), which are generated by the chemical reaction of specific PM-compositions from anthropogenic sources [13,21]. Both *in vivo* and *in vitro* studies have demonstrated that the chemical composition of PM could induce oxidative injury, inflammation, fibrosis, and cytotoxicity in the lung [19,22,23]. It was found that redox-active transition metals (e.g., Fe, Cu, Cr, Ni, and Zn) undergoing redox cycling directly are associated with ROS generation [24], whereas organic compounds, such as polyaromatic hydrocarbons (PAH), indirectly affect the production of ROS [25]. The ability of chemical composition to induce oxidant radical generation may provide evidence to compare the toxic potential of PM.

In the present study, to improve knowledge about the role played by metals coated onto PM in lung cytotoxicity, we investigated the capacity of various size-fractionated PM collected from an urban traffic site (Beijing City) and an industrial site (Anshan City) to induce ROS generation in human bronchial epithelial BEAS-2B cells. Moreover, the effect of PM-induced ROS generation on cell viability was evaluated. The correlation analysis of the chemical compositions of PM and redox activity or cell viability were also performed. Furthermore, we assessed the effect of metal-chelation with desferoxamine (DFO) on ROS generation and cell viability.

## 2. Materials and Methods

### 2.1. Materials

Human bronchial epithelial cell line BEAS-2B was obtained from the China Center for Type Culture Collection (Shanghai, China). Dulbecco’s modified Eagle’s medium (DMEM) was purchased from Sigma-Aldrich (St. Louis, MO, USA). Penicillin and streptomycin were purchased from Gibco (Grand Island, NY, USA). 2’,7’-dichlorodihydrofluorescein diacetate (DCFH-DA) were purchased from Sigma-Aldrich. 3-(4,5-dimethylthiazol-2-yl)-5-(3-carboxy-methoxyphenyl)-2-(4-sulfo-phenyl)-2H-tetra-zolium salt (MTS) was purchased from Promega (Madison, WI, USA). Fetal bovine serum was purchased from PAA (Linz, Austria). Ninety-six-well plates and cell culture dishes were purchased from Costar (Cambridge, MA, USA). A ToxinSensor chromogenic limulus amebocyte lysate endotoxin assay kit was purchased from GenScript (Piscataway Township, NJ, USA). Desferrioxamine (DFO) was purchased from Sigma-Aldrich.

### 2.2. Sampling and Particle Preparation

PM of three aerodynamic diameter ranges (<1, 1–2.5 and 2.5–10 µm) were collected at two different sites: (i) An urban ambient site (u) was chosen on a rooftop (about 13 m aboveground) in the Yuquan campus of the University of Chinese Academy Sciences (UCAS), close to the Western Fifth-ring road of Beijing City, in March–July 2013. There is high traffic flow and a high density of population in the daytime. The UCAS campus is surrounded by some institutes and residential areas. Large industrial and thermoelectric plants were absent; the distance of the sampler from the main road was 10 m; (ii) The steel factory ambient site (s) is located in the industrial area of Anshan City, China (about 670 km away from Beijing). Air sampling was performed in November–December 2014, with sunny weather. PM was collected on 90 mm Teflon filters (diameter = 90 mm, Whatman, Piscataway, NJ, USA) by medium-volume PM samplers (Wuhan Tianhong Intelligence Instrumentation Facility, model TH-150D II, flow rate: 100 L/min). Before and after the sampling, the Teflon filters were equilibrated in conditions of 30% relative humidity and 25 °C room temperature for over 48 h and then weighted on a high-precision microbalance (Mettler Toledo, OH, USA) to measure the collected PM. All sampled filters were stored in the dark at −20 °C before further chemical and physical characterization. Unexposed filters (blank samples) were prepared using the same method except for sampling and were used as a control in all experiments.

Particles on Teflon filters were extracted according to the method of Imrich *et al.* [26]. Briefly, PM samples were extracted from the sampled filters by immersing them in deionized water (18.2 MΩ/cm) and then sonicating them for 30 min in a water-bath sonicator (KQ-700V, 700W). PM samples were then stored at −80 °C until use. Blank samples were processed simultaneously with the PM samples and used as a control in all experiments. To adjust the concentration of PM preparations, 100 µL aliquots of PM samples were placed on filters and air-dried. The samples and filters were weighed on a microbalance (Mettler Toledo, Switzerland). PM samples were prepared in deionized water at 5 mg/mL and sonicated for 1 min prior to use.

### 2.3. PM Physical and Chemical Characterization

The size distribution of various uPM and sPM was measured using scanning electron microscopy (SEM, JEOL JSM-6700F, Tokyo, Japan) as described by Deng *et al.* [19]. Prior to analysis, PM was suspended in an n-hexane solution with a assistance of ultrasonic treatment, and the suspended particles was then filtered through a nucleopore filter to obtain well distribution and dispersed PM, without agglomerates. The filter was then carbon coated and measured using automatic mode.

The size distribution of different PM in suspension were analyzed using Nano-Zetasizer (1000 HS, Malvern Instruments Ltd., Malvern, UK) based on the dynamic light scattering measurement technique. Before analysis, particles were first suspended in a serum-free culture medium and sonicated with an ultrasonic processor (VCX130, Sonics, Newtown, CT, USA) for 30 s at 40 W in a bath to disperse evenly.

PM samples from both sampling sites were chemically characterized for elemental carbon (EC) and organic carbon (OC), water soluble inorganic ions, and inorganic elements. All chemical components were analyzed for each single sample and the pooled sample. For metal analysis, the analytical procedure for inorganic element determination comprised the acidic total digestion (HNO_3_:HF = 7:3) of collected PM samples (1 mg) and the subsequent analysis of 20 elements by inductively coupled plasma-mass spectrometry (ICP-MS, Elemental X7, Thermo, Waltham, MA, USA). Element stock solutions were prepared, containing 1 µg/mL of each analyte (Ti, V, Cr, Mn, Co, Ni, Cu, As, Sr, Cd, Pb, Zn, Fe, Ca, K, Mg, and Na). The multi-element working standard solution was prepared (using the reagent blank solution as diluent) from the abovementioned stock solution to span the concentration range from 10 to 100 ng/mL. At these concentration levels, all elements could be mixed together. In (20 ng/mL) and Bi (20 ng/mL) were used as the internal standards. The detection limit for each element in solution was calculated as three times the standard deviation of the ion counts obtained for the sample blank (measured for 10 replicate determinations), divided by the sensitivity of 10 ng/mL multi-element standard solutions. For Co, V, Cr, Ni, Ti, Cu, Zn, Sr, Mo, Cs, Ba, and Pb the detection limits ranged from 0.01 to 0.2 ng/mL. The water-soluble inorganic components (e.g., SO_4_^2−^, NO_3_^−^, NH_4_^+^, and Cl^−^) were extracted in ultra-pure water for 30 min using an ultrasonic bath, filtered (0.45 mm PTFE filters), and identified by ionic chromatography (Dionex-600, Sunnyvale, CA, USA). The detection limits in µg/mL were 0.01 for Cl^−^, 0.03 for NO_3_^−^, 0.02 for SO_4_^2−^, 0.02 for NH_4_^+^, and 0.01 for Ca^2+^, Mg^2+^, Na^+^, and K^+^. Elemental and organic carbon in PM samples were measured on-filter using a thermal-optical analyzer (Sunset Laboratory, Inc., Hillsborough, NC, USA) according to the method of Zhang *et al.* [27]. Endotoxin levels in PM were determined by Limulus amebocyte lysate assay kits following the manufacturer’s instructions.

### 2.4. Cell Culture and PM Treatment

Human bronchial epithelial BEAS-2B cells were maintained in high glucose DMEM supplemented with 10% (*v*/*v*) FBS, 100 IU/mL penicillin and 100 µg/mL streptomycin in a 5% CO_2_ humidified atmosphere at 37 °C. All cell exposure experiments were performed at 80%–90% of cell confluence, with viability ≥90% determined by trypan blue staining.

The cells were then harvested using 0.25% trypsin and were sub-cultured into 24-well plates or 96-well plates according to selection of experiments. Cells were allowed to attach to the surface in DMEM supplemented 10% FBS for 24 h prior to treatment. Then, the culture medium was replaced with a serum-free medium. The cells were exposed to freshly dispersed PM preparations at a final concentration of 100 µg/mL for 24 h [19,28].

### 2.5. MTS Assay

MTS assay was carried out to assess the cell viability after exposure to PM according to the method of Malich *et al.* [29]. Briefly, BEAS-2B cells were plated into 96-well plates at a density of 1.0 × 10^4^ cells per well in 100 µL medium and cultured for 24 h. After incubation, BEAS-2B cells were treated with 0 or 100 µg/mL PM for 24 h. After exposure, 10 µL of MTS reagent was added directly to the wells and cell plates were incubated at for 1 h. The absorbance was quantified by a microplate spectrophotometer (Thermo MK3, MA, USA) at a wavelength of 492 nm. The viability of the treated cells was presented as a percentage of untreated cells, which was assumed to be 100%.

### 2.6. Reactive Oxygen Species Assay

The level of intracellular reactive oxygen species (ROS) in BEAS-2B cells was determined by measuring the oxidative conversion of DCFH-DA to fluorescent compound dichlorofluorescein (DCF). DCFH-DA, dissolved in ethanol, was added to cell culture at a final concentration of 40 µM for 30 min at 37 °C. BEAS-2B cells (2 × 10^5^ cells) were washed with PBS and then exposed to 0 or 100 µg/mL of PM for 3 h, respectively. After exposure, the cells were lysed with 400 mM of NaOH. The total green fluorescence intensity was detected in a fluorescence multi-well plate reader (TriStar LB 941, Berthold, Bad Wildbad, Germany) with excitation and emission wavelengths of 485 nm and 535 nm. Results were measured as the fluorescence intensity change of untreated cells.

### 2.7. DFO Administration in MTS and ROS Assay

To further investigate the effects of metal elements on the cell viability and ROS generation, the chelator desferoxamine (DFO) was used to chelate the metal ions and to inhibit metal-mediated secondary oxidant generation. DFO is a metal chelator with an affinity for iron several logs of magnitude stronger than other metals’ [30]. Complexation with DFO was carried out according to the method of Shafer *et al.* [30]. The PM suspensions were processed by adding 50 µL of 1000 µM DFO solution to 950 µL of PM sample (concentration of DFO is 50 µM). DFO was allowed to equilibrate for a minimum of 30 min in the samples and then the samples were immediately prepared for the ROS assay and MTS assay.

### 2.8. Statistical Analysis

Data were expressed as mean ± standard deviation (SD) of three independent experiments. Data were analyzed using one-way analysis of variance (ANOVA) followed by *post* *hoc* comparisons using the Tukey’s multiple paired comparison test. Statistical significance was set at *p* < 0.05. The correlation analysis was performed using Pearson’s test to assess associations between ROS generation/cell viability and chemical components of PM. All individual measurements for data of all particle sizes were combined and reflected the pooled association. GraphPad Prism 5.0 was used for all the statistical analyses.

## 3. Results

### 3.1. PM Physical and Chemical Characteristics

**Figure 1:**
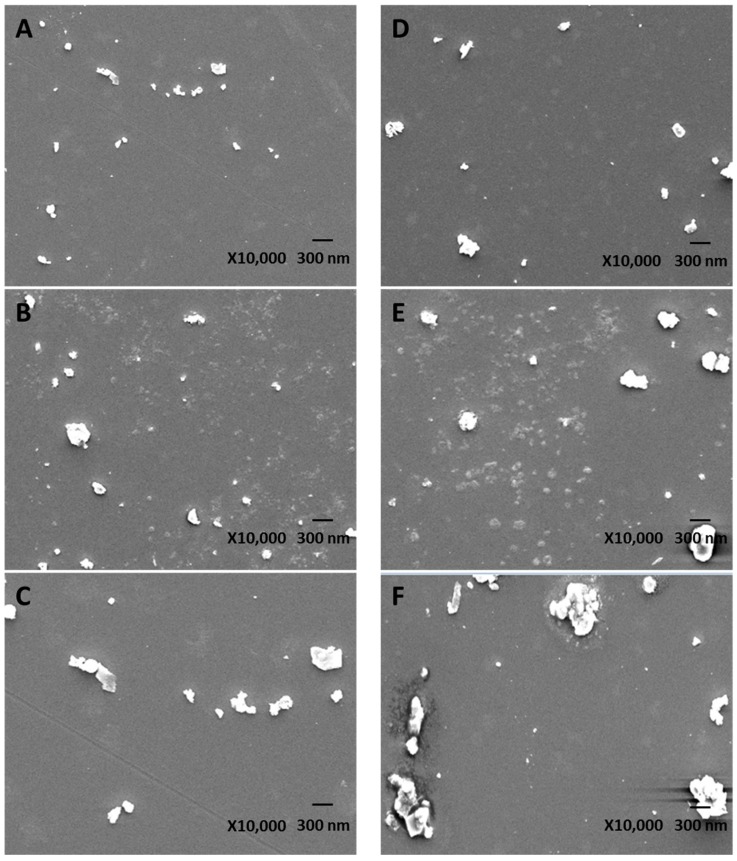
Scanning electron microscope (SEM) images of the different PM (Bar, 300 nm; magnification 10,000×): (**A**) uPM_1.0_; (**B**) uPM_1.0–2.5_; (**C**) uPM_2.5–10_; (**D**) sPM_1.0_; (**E**) sPM_1.0–2.5_; and (**F**) sPM_2.5–10_.

The SEM images of uPM_1.0_, uPM_1.0–2.5_, uPM_2.5–10_, sPM_1.0_, sPM_1.0–2.5_, and sPM_2.5–10_ are shown in Figure 1. Size distributions of the PM fraction collected in two different locations are shown in Table 1. The mean hydrated diameter of uPM_1.0_, uPM_1.0–2.5_, and uPM_2.5–10_ was 0.22, 0.36, and 0.49 µm, respectively. The mean hydrated diameter of sPM_1.0_, sPM_1.0–2.5_, and sPM_2.5–10_ was 0.29, 0.53, and 0.62 µm, respectively. The diameters of sPM_1.0_, sPM_1.0–2.5_, and sPM_2.5–10_ sampled from the steel factory site were larger than those of the urban size-fractionated PM.

Significant differences among the three size fractions were observed for all metals (Table 2). In uPM, the concentrations of Al, Ca, Fe, Mg, Na, Ti, V, Mn, Co, and Ni increased as the particle size increased. For Cu, As, Cd, Pb, and Zn, the highest concentrations were observed in the uPM_1.0_. Compared to the uPM_1.0_, Ca, Fe, K, Na, Cr, Mn, Ni, Cu, As, Cd, Cs, Pb, and Zn were the most abundant elements in sPM_1.0_. The concentrations of these elements in sPM_1.0_ decreased as the particle sized increased. Moreover, the concentrations of Mn, Cu, Fe, Pb, and Zn in sPM_1.0_ were 24, 14, 11, 9, 7, and 4 times higher than those in uPM_1.0_, respectively (Table 2). For Al, Ti, Co, Sr, and Ba, the highest concentrations occurred in the fraction with size between 2.5 and 10 µm. In addition, the concentrations of endotoxin in the PM were 3.32 ± 1.07 (uPM_1.0_), 21.17 ± 1.88 (uPM_1.0–2.5_), 133.25 ± 3.57 (uPM_2.5–10_), 6.53 ± 2.98 (sPM_1.0_), 43.12 ± 2.16 (sPM_1.0–2.5_), and 134.86 ± 2.64 (sPM_2.5–10_) EU/mg, respectively. It was apparent that endotoxins generally increased as particle size increased, which tended to be associated with coarse particles. No significant difference between uPM_2.5–10_ and sPM_2.5–10_ was observed for endotoxin. However, endotoxins in the sPM_1.0_ and sPM_1.0–2.5_ were about double those in uPM_1.0_ and uPM_1.0–2.5_.

As shown in Table 3, significant differences among the three size fractions can be seen for all inorganic ions. The concentrations of SO_4_^2−^, NO_3_^−^, NH_4_^+^, and Cl^−^ in both uPM and sPM decreased as the particle sized increased. For SO_4_^2−^, NO_3_^−^, and NH_4_^+^, the highest concentrations occurred in the uPM_1.0_ and sPM_1.0_. The concentrations of SO_4_^2−^, NO_3_^−^, NH_4_^+^, and Ca^2+^ in uPM_1.0_, uPM_1.0–2.5_, and uPM_2.5–10_ were significantly higher than those in sPM_1.0_, sPM_1.0–2.5_, and sPM_2.5–10_. However, the concentrations of Cl^−^, K^+^, Na^+^, and Mg^2+^ in uPM_1.0_ and uPM_1.0–2.5_ were lower than those in sPM_1.0_ and sPM_1.0–2.5_. These results suggested that urban fine PM was from secondary aerosol formation [31].

Table 4 showed that the concentrations of OC and EC in uPM decreased as the particle size increased. The highest concentrations of OC and EC occurred in uPM_1.0_. However, the concentrations of OC and EC in uPM_1.0–2.5_ and uPM_2.5–10_ were lower than those in sPM_1.0–2.5_ and sPM_2.5–10_. Moreover, the OC/EC ratio in both uPM and sPM increased as the particle size increased.

### 3.2. Effect of PM on Cell Viability in BEAS-2B Cells

**Figure 2:**
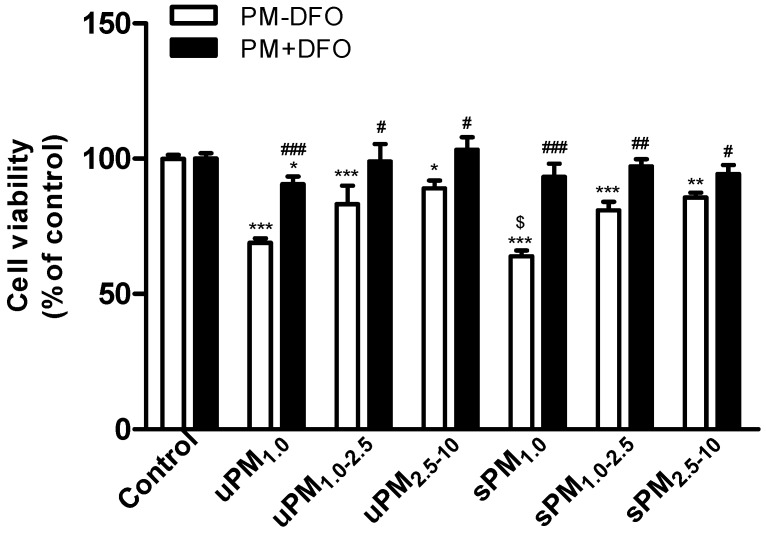
Effects of PM and DFO-treated PM on the cell viability in BEAS-2B cells. BEAS-2B cells were exposed to 100 μg/mL of uPM_1.0_, uPM_1.0–2.5_, uPM_2.5–10_, sPM_1.0_, sPM_1.0–2.5_, and sPM_2.5–10_ for 24 h with or without 50 μM DFO pretreatment. The cell viability was determined by MTS assay. Control cells were treated with and without DFO in the absence of PM. Values are represented as mean ± SD of three independent experiments. * *p* < 0.05, ** *p* < 0.01, and *** *p* < 0.001 *vs.* the untreated control cells; ^#^ *p* < 0.05, ^##^ *p* < 0.01, and ^###^ *p* < 0.001 *vs.* DFO-untreated PM; ^$^ *p* < 0.05 *vs.* the uPM-treated cells.

BEAS-2B cells were exposed to 100 µg/mL of three size fractions of uPM and sPM for 24 h. The cell viability was examined by MTS assay. As shown in Figure 2, the cell viability significantly decreased after exposure to three fractions of uPM and sPM compared with the unexposed control cells. Moreover, the cell viability in sPM_1.0_-treated cells was significantly lower than that in uPM_1.0_-treated cells (*p* < 0.05), indicating that sPM_1.0_ exhibited higher toxicity than uPM_1.0_. Moreover, the cell viability in uPM- or sPM-treated cells increased after pretreatment of various size-fractionated of uPM or sPM with the metal chelator DFO.

### 3.3. Effect of PM on ROS Generation in BEAS-2B Cells

**Figure 3:**
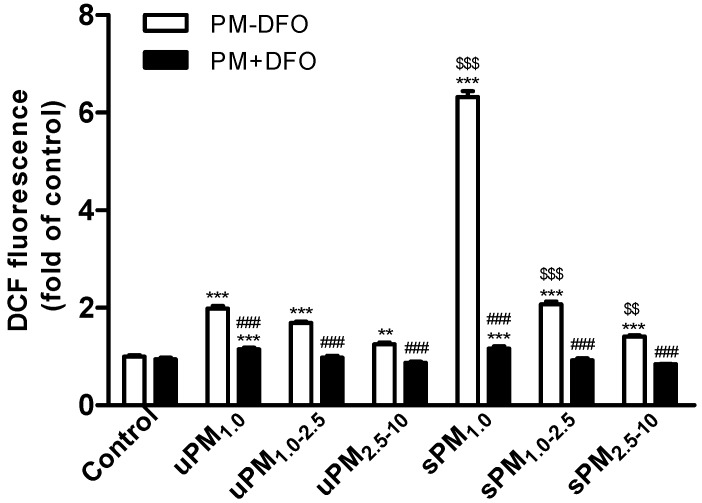
Effects of PM and DFO-treated PM on intracellular ROS generation in BEAS-2B cells. BEAS-2B cells were exposed to 100 μg/mL of uPM_1.0_, uPM_1.0–2.5_, uPM_2.5–10_, sPM_1.0_, sPM_1.0–2.5_, sPM_2.5–10_ for 3 h with or without 50 μM DFO pretreatment. The ROS levels were determined by measuring the oxidative conversion of DCFH-DA to DCF. Control cells were treated with and without DFO in the absence of PM. Results were measured as mean fluorescence (arbitrary units, AU). Values are represented as mean ± SD of three independent experiments. ** *p* < 0.01 and *** *p* < 0.001 *vs.* the untreated control cells; ^###^ *p* < 0.001 *vs.* DFO-untreated PM; ^$$^ *p* < 0.01 and ^$$$^ *p* < 0.001 *vs.* the uPM-treated cells.

The DCF fluorescence intensity in BEAS-2B cells was detected after exposed to 100 µg/mL of uPM_1.0_, uPM_1.0–2.5_, uPM_2.5–10_, sPM_1.0_, sPM_1.0–2.5_, and sPM_2.5–10_ for 3 h. As shown in Figure 2, compared with the unexposed control cells, the level of ROS generation was highest in uPM_1.0_, followed by uPM_1.0–2.5_ and uPM_2.5–10_. Similar observations regarding the effect of particle size on ROS generation were also made with sPM. However, the highest ROS generation per mass of PM was associated with the sample collected in the steel factory. The level of ROS in sPM_1.0_-treated cells was triple that in uPM_1.0_-treated cells, suggesting that the metal elements in sPM may be predominantly responsible for ROS generation. Pretreatment of uPM or sPM with DFO was effective at decreasing ROS generation (Figure 3). In particular, the level of ROS in sPM_1.0_-treated cells significantly decreased compared with that in uPM_1.0_-treated cells.

### 3.4. Correlation of Cell Viability and ROS Generation with Different PM Compositions

In our exploratory data analysis, we attempted to identify the correlations between the cell viability and ROS generation measured in PM-treated in cells and their chemical compositions. Table 5 and Table 6 show the Pearson correlation coefficients (*r*) and the associated coefficient of significance (*p*). All particle size ranges (PM_1.0_, PM_1.0–2.5_, and PM_2.5–10_) have been combined in this correlation. As shown in Table 5, there was a strong negative correlation between the cell viability and EC, SO_4_^2−^, NH_4_^+^, Cl^−^, As, Cd, and Zn. However, the cell viability from these samples showed a strong positive correlation with Al, Ti, and Sr. No significant correlation of ROS generation with OC, EC, SO_4_^2−^, NO_3_^−^, NH_4_^+^, Al, Ca, Mg, Ti, V, and Co was measured (Table 6). However, the results from Table 6 showed a high degree of correlation between Cl^−^, Fe, K, Na, Cr, Mn, Ni, Cu, As, Cd, Cs, Pb, and Zn and ROS generation. In addition, a significantly negative correlation at *p* = 0.05 with Sr and Ba was observed.

## 4. Discussion

In this study, we used the human bronchial epithelial cell line BEAS-2B as a model to investigate the effects of the chemical composition of various size-fractionated PM on cytotoxicity and intracellular oxidant activity. Previous studies have reported that the chemical composition of PM may be related to the size-fractions and sources [32,33]. It has been demonstrated that the coarse fraction of PM is richer in crustal elements, whereas the fine fraction of PM is composed mainly of combustion components [8,15]. In the present study, various size-fractioned PM (PM_1.0_, PM_1.0–2.5_, and PM_2.5–10_) from an urban site and a steel-factory site were collected and chemically characterized. Differences in chemical composition between urban and steel-factory PMs were related to differences in the size fractions of uPM and sPM. The concentrations of SO_4_^2−^, NO_3_^−^, NH_4_^+^, OC, and EC in the uPM were higher than those in sPM. This may reflect its multiple sources: coal combustion, motor vehicle exhaust, and biomass burning in Beijing [19]. Among 12 detected inorganic elements including natural sourced elements (Ca, Mg, K, Na, Ti, *etc.*) and conventionally anthropogenic inorganic elements (Fe, Mn, Ni, Cr, Pb, Zn, Cu, *etc.*), relatively higher levels of Fe, Cu, Mn, Pb, and Zn were determined in the PM_1.0_ and PM_1.0–2.5_ than in the PM_2.5–10_. This observation is in agreement with the previous report that metals are more abundant in the fine fraction than in the coarse fraction [34]. In addition, we observed that the concentrations of Ca, Fe, K, Na, Cr, Mn, Ni, Cu, As, Cd, Cs, Pb, and Zn were higher in sPM_1.0_ than those in uPM_1.0_. In terms of concentration values, the concentrations of Mn, Cu, Fe, Pb and Zn in sPM_1.0_ were 24, 14, 11, 9, 7, and 4 times higher than those in uPM_1.0_. It has been reported that these metals are often associated with industrial emissions [35]. Among the trace elements, the most abundant element in uPM and sPM was Fe, which may be derived from car exhaust and other urban combustion sources as well as industrial activities [36]. Moreover, we found that endotoxins generally increased as particle size increased and were enriched in coarse particles, which is similar to the results reported in the literature [37]. The concentrations of endotoxin in the uPM_2.5–10_ (133.25 ± 3.57 EU/mg) and sPM_2.5–10_ (134.86 ± 2.64 EU/mg) were much higher than those in PM_10_ (20.1–49.3 EU/mg) collected from Mexico City [38].

Several studies have demonstrated that PM toxicity is dependent on both size and composition [37,38]. Fine PM have higher cytotoxicity when compared with coarse PM [7,37]. However, coarse PM is found to be more toxic than fine PM due to high levels of endotoxin and transition metals [38,39]. In the present study, we found that different size-fractioned uPM and sPM caused a significant decrease in cell viability. No significant difference between PM_1.0–2.5_ and PM_2.5–10_ was observed (Figure 2), although PM_1.0_ was more toxic than PM_1.0–2.5_ and PM_2.5–10_, especially sPM_1.0_. These results are in agreement with the report that the toxicity difference between fine and coarse PM depended on sampling location [40]. In addition, we found that in uPM, metal concentrations including Fe increased as the particle size increased. However, cell viability was the lowest in uPM_1.0_. Significant difference among the size fractions can be seen for all metals (Table 2). The highest concentrations of Cu, As, Cd, Pb, and Zn occurred in the uPM_1.0_, similar to the results reported in the literature [9]. Alfaro-Moreno *et al.* found that Ni and Zn are responsible for the loss of viability induced by PM [36]. Al, Fe, Zn, Ba, and Mn of fine PM decrease cell viability in human lung epithelial A549 cells [37]. However, it was found that Zn and Cu were more toxic to cells than Ni, Fe, Pb, or V [41]. Further analysis showed that the cell viability was negatively correlated with the EC, SO_4_^2−^, NH_4_^+^, Cl^−^, Al, Ti, As, Sr, Cd, and Zn content of the PM samples. This observation is consistent with a previous report of a negative correlation between cell viability reduction and the elements As, Zn, Cr, Cu, and Mn [42]. A significant correlation was found between Al, As, Cr, Cu, and Zn of fine particulate matter (PM_1_ and PM_2.5_) and the biological response in human lung epithelial cells (A549) [42]. This suggests that the cytotoxic effects of the chemical composition of various PM size fractions differ greatly [40].

It has been identified that PM could provoke intracellular ROS generation [43,44]. ROS overproduction by redox-active transition metals, water soluble inorganic ions, carbonaceous fractions, and polycyclic aromatic hydrocarbons of PM_2.5_ have been described [45,46,47,48,49]. In the present study, we found that exposure to size-fractioned uPM and sPM led to a significant increase in intracellular ROS generation. Among all PM samples, sPM_1.0_ was the strongest ROS inducer. We found that the increase of ROS in sPM_1.0_-treated cells was triple that in uPM_1.0_-treated cells. Intracellular ROS generation in this study was positively correlated with Fe, Cr, Mn, Ni, Cu, As, Sr, Cd, Cs, Ba, Pb, and Zn. Among these trace elements, transition metals are responsible for the oxidant activity of PM and result in ROS generation via Fenton-type reactions [24]. Cd, Pb, and Zn can induce oxidative stress via depletion of cellular antioxidant pools (e.g., glutathione), and increase lipid peroxidation [30]. Moreover, pretreatment with DFO significantly decreased uPM- and sPM-induced intracellular ROS generation, suggesting that Fe accounts for a large majority of PM-induced ROS activity.

## 5. Conclusions

In conclusion, our results demonstrate that the metals absorbed on the different size-fractionated PM have more potential to trigger cellular oxidant radical generation, especially the small particles from the steel factory. These results indicate that variations in the chemical composition of different size-fractionated PM cause changes in the toxicological response. Moreover, we found that the cytotoxicity and oxidant radical generation of bronchial epithelial cells are significantly different after exposure to equal mass concentrations of urban and industrial PM. These observations also confirm the hypothesis that particle composition and source constitute an important factor in PM-induced toxicity. Further studies of source-related PM toxicity are essential for the understanding of toxicological mechanisms and an adapted risk assessment.

## Figures and Tables

**Table 1:** Diameter of different size fractions of PM.

Sampling Site	Sampling Period	Particles	Aerodynamic Diameter (µm)	Hydrated Diameter (µm)
Urban (Beijing)	March–July	uPM_1.0_	<1.0	0.22 (0.15–0.31)
		uPM_1.0–2.5_	1.0–2.5	0.36 (0.23–0.55)
		uPM_2.5–10_	2.5–10	0.49 (0.36–0.64)
Steel factory (Anshan)	November–December	sPM_1.0_	<1.0	0.28 (0.20–0.36)
		sPM_1.0–2.5_	1.0–2.5	0.53 (0.30–0.86)
		sPM_2.5–10_	2.5–10	0.62 (0.41–0.99)

**Table 2:** Element analysis in different size fractions of PM (µg/mg).

Element	Urban (Beijing)			Steel Factory (Anshan)		
	uPM_1.0_	uPM_1.0–2.5_	uPM_2.5–10_	sPM_1.0_	sPM_1.0–2.5_	sPM_2.5–10_
Al	18.71 ± 0.12	37.56 ± 0.14	54.91 ± 0.38	14.00 ± 0.11	32.18 ± 0.01	44.91 ± 0.30
Ca	23.23 ± 0.22	72.64 ± 0.33	97.74 ± 0.47	47.57 ± 0.28	32.62 ± 0.24	34.62 ± 0.28
Fe	12.68 ± 0.10	30.30 ± 0.19	32.72 ± 0.20	142.43 ± 0.30	51.81 ± 0.19	34.41 ± 0.14
K	22.55 ± 0.11	12.68 ± 0.09	16.56 ± 0.12	64.90 ± 0.15	11.06 ± 0.03	12.82 ± 0.14
Mg	6.36 ± 0.03	15.19 ± 0.09	24.27 ± 0.11	22.63 ± 0.08	18.46 ± 0.06	22.56 ± 0.08
Na	5.74 ± 0.03	8.39 ± 0.04	12.75 ± 0.07	54.03 ± 0.16	10.12 ± 0.05	9.53 ± 0.04
Ti	0.69 ± 0.02	2.32 ± 0.06	3.79 ± 0.08	1.19 ± 0.003	3.28 ± 0.005	4.87 ± 0.005
V	0.05 ± 0.001	0.08 ± 0.001	0.10 ± 0.002	0.11 ± 0.0004	0.08 ± 0.001	0.11 ± 0.001
Cr	0.13 ± 0.003	0.15 ± 0.003	0.14 ± 0.004	0.84 ± 0.001	0.24 ± 0.0004	0.17 ± 0.0002
Mn	0.81 ± 0.02	0.85 ± 0.02	1.09 ± 0.02	19.81 ± 0.08	2.42 ± 0.02	1.22 ± 0.01
Co	0.009 ± 0.002	0.02 ± 0.003	0.02 ± 0.003	0.02 ± 0.002	0.03 ± 0.004	0.03 ± 0.004
Ni	0.05 ± 0.001	0.07 ± 0.001	0.07 ± 0.001	0.20 ± 0.001	0.11 ± 0.001	0.10 ± 0.001
Cu	0.51 ± 0.008	0.36 ± 0.005	0.19 ± 0.001	7.19 ± 0.02	0.56 ± 0.007	0.29 ± 0.004
As	0.17 ± 0.005	0.11 ± 0.004	0.03 ± 0.002	0.51 ± 0.003	0.13 ± 0.003	0.06 ± 0.001
Sr	0.30 ± 0.005	0.36 ± 0.004	0.39 ± 0.005	0.17 ± 0.0002	0.42 ± 0.007	0.45 ± 0.006
Cd	0.03 ± 0.001	0.01 ± 0.0003	0.003 ± 0.0001	0.05 ± 0.0003	0.02 ± 0.001	0.01 ± 0.0001
Cs	0.01 ± 0.0001	0.01 ± 0.0001	0.005 ± 0.0001	0.07 ± 0.0004	0.009 ± 0.0001	0.01 ± 0.0001
Ba	0.85 ± 0.007	0.88 ± 0.007	0.81 ± 0.006	0.40 ± 0.004	1.22 ± 0.01	1.20 ± 0.01
Pb	0.97 ± 0.008	0.39 ± 0.004	0.05 ± 0.001	8.50 ± 0.06	0.82 ± 0.004	0.35 ± 0.003
Zn	3.39 ± 0.05	1.38 ± 0.03	0.52 ± 0.008	13.25 ± 0.03	2.82 ± 0.02	0.81 ± 0.01

**Table 3:** Inorganic ions in different size fractions of PM (µg/mg).

Ions	Urban (Beijing)			Steel Factory (Anshan)		
	uPM_1.0_	uPM_1.0–2.5_	uPM_2.5–10_	sPM_1.0_	sPM_1.0–2.5_	sPM_2.5–10_
SO_4_^2−^	141.45 ± 10.05	70.50 ± 5.27	39.16 ± 3.36	125.04 ± 7.83	45.92 ± 4.82	25.26 ± 0.82
NO_3_^−^	114.94 ± 9.86	85.71 ± 6.13	32.08 ± 1.52	80.79 ± 8.60	48.71 ± 3.52	23.95 ± 1.05
NH_4_^+^	61.50 ± 8.62	9.10 ± 0.88	1.55 ± 0.02	36.40 ± 5.26	7.38 ± 0.85	1.12 ± 0.03
Cl^−^	31.95 ± 3.26	10.95 ± 1.05	9.59 ± 0.89	48.58 ± 4.75	17.46 ± 1.24	7.18 ± 0.34
Ca^2+^	15.39 ± 0.58	46.81 ± 2.23	36.65 ± 1.37	10.25 ± 0.27	39.27 ± 1.93	30.15 ± 0.61
K^+^	13.15 ± 1.07	2.62 ± 0.17	1.45 ± 0.11	23.31 ± 1.58	3.73 ± 0.21	1.38 ± 0.05
Na^+^	4.55 ± 0.16	3.99 ± 0.14	4.30 ± 0.19	17.49 ± 0.89	8.35 ± 0.54	3.19 ± 0.10
Mg^2+^	2.64 ± 0.23	3.27 ± 0.30	2.49 ± 0.26	4.84 ± 0.51	4.05 ± 0.36	0.87 ± 0.09

**Table 4:** OC/EC in different size fractions of PM (µg/mg).

OC/EC	Urban (Beijing)			Steel Factory (Anshan)		
	PM_1.0_	PM_1.0–2.5_	PM_2.5–10_	PM_1.0_	PM_1.0–2.5_	PM_2.5–10_
OC	108.62 ± 6.61	60.99 ± 4.96	56.53 ± 0.18	67.61 ± 1.18	82.49 ± 10.59	71.84 ± 1.45
EC	51.96 ± 6.64	20.44 ± 1.35	11.67 ± 0.37	34.18 ± 4.13	26.48 ± 2.27	14.03 ± 3.61
OC/EC	2.09 ± 0.40	2.98 ± 0.44	4.85 ± 0.17	1.98 ± 0.21	3.12 ± 0.23	5.12 ± 1.71

**Table 5:** Correlation of cell viability with different PM compositions.

Components	Correlation of Cell Viability		
	r	*p*	Significance
OC	−0.5080	0.3036	
EC	−0.8467	0.0335	*
SO_4_^2−^	−0.9181	0.0098	**
NO_3_^−^	−0.4664	0.3511	
NH_4_^+^	−0.8686	0.0248	*
Cl^−^	−0.9698	0.0014	**
Al	0.9702	0.0013	**
Ca	0.5266	0.2832	
Fe	−0.5922	0.2155	
K	−0.8005	0.0557	
Mg	0.3924	0.4416	
Na	−0.6420	0.1693	
Ti	0.8591	0.0284	*
V	0.2356	0.6531	
Cr	−0.7017	0.1202	
Mn	−0.7140	0.1110	
Co	0.4167	0.4112	
Ni	−0.5377	0.2712	
Cu	−0.7493	0.0864	
As	−0.8739	0.0228	*
Sr	0.8759	0.0221	*
Cd	−0.9525	0.0033	**
Cs	−0.7699	0.0733	
Ba	0.6471	0.1648	
Pb	−0.7831	0.0655	
Zn	−0.8500	0.0320	*

* and ** indicate statistical significance at *p* < 0.05 and *p* < 0.01.

**Table 6:** Correlation of ROS generation with different PM compositions.

Compositions	Correlation of ROS Generation		
	r	*p*	Significance
OC	−0.0710	0.8936	
EC	0.3819	0.4551	
SO_4_^2−^	0.6250	0.1846	
NO_3_^−^	−0.0508	0.9238	
NH_4_^+^	0.4553	0.3642	
Cl^−^	0.8976	0.0152	*
Al	−0.7245	0.1034	
Ca	−0.1358	0.7975	
Fe	0.9349	0.0062	**
K	0.9833	0.0004	***
Mg	0.1795	0.7336	
Na	0.9655	0.0018	**
Ti	−0.5826	0.2249	
V	0.3212	0.5347	
Cr	0.9768	0.0008	***
Mn	0.9844	0.0004	***
Co	−0.1077	0.8391	
Ni	0.8871	0.0184	*
Cu	0.9933	<0.0001	***
As	0.9901	0.0001	***
Sr	−0.9073	0.0125	*
Cd	0.9166	0.0101	*
Cs	0.9951	<0.0001	***
Ba	−0.8200	0.0457	*
Pb	0.9962	<0.0001	***
Zn	0.9923	<0.0001	***

*, ** and *** indicate statistical significance at *p* < 0.05, *p* < 0.01 and *p* < 0.001.
